# Respiratory Viruses in Solid Organ Transplant Recipients

**DOI:** 10.3390/v13112146

**Published:** 2021-10-25

**Authors:** Roni Bitterman, Deepali Kumar

**Affiliations:** Ajmera Transplant Centre, University Health Network, Toronto, ON M5G 2N2, Canada; roni.bitterman@uhn.ca

**Keywords:** vaccine, prevention, lung transplant, influenza, RSV, metapneumovirus, coronavirus, parainfluenza

## Abstract

Solid organ transplantation is often lifesaving, but does carry an increased risk of infection. Respiratory viral infections are one of the most prevalent infections, and are a cause of significant morbidity and mortality, especially among lung transplant recipients. There is also data to suggest an association with acute rejection and chronic lung allograft dysfunction in lung transplant recipients. Respiratory viral infections can appear at any time post-transplant and are usually acquired in the community. All respiratory viral infections share similar clinical manifestations and are all currently diagnosed using nucleic acid testing. Influenza has good treatment options and prevention strategies, although these are hampered by resistance to neuraminidase inhibitors and lower vaccine immunogenicity in the transplant population. Other respiratory viruses, unfortunately, have limited treatments and preventive methods. This review summarizes the epidemiology, clinical manifestations, therapies and preventive measures for clinically significant RNA and DNA respiratory viruses, with the exception of SARS-CoV-2. This area is fast evolving and hopefully the coming decades will bring us new antivirals, immunologic treatments and vaccines.

## 1. Introduction

There are numerous respiratory viruses that have a significant impact on the health of immunocompromised organ transplant recipients, and new viruses and serotypes are continuously being discovered. In this review we will cover both RNA and DNA respiratory viruses, including influenza virus, respiratory syncytial virus (RSV), human metapneumovirus (HMPV), parainfluenza virus (PIV), rhinovirus, coronavirus (CoV), adenovirus, bocavirus and KI and WU polyomaviruses, specifically as they relate to organ transplant recipients. Although all organ types will be discussed, the vast majority of data comes from studies of lung transplant recipients. Although hematopoietic stem cell transplantation is not a focus of this review, data from this population will be used where it informs the management of organ transplant recipients. The recent SARS-CoV-2 pandemic virus will be discussed as part of other reviews in this series.

In organ transplant recipients, the incidence of respiratory viral infections in transplant ranges between 0.76–0.91 episodes per patient-year [1,2,3], although part of the studies also included asymptomatic infections. The incidence appears to be higher among lung transplant recipients (LTRs) [4], but is not affected by time from transplant [1]. Respiratory viruses are present throughout the year, but there is a higher incidence in the autumn and winter, and patterns differ between viruses [2]. All respiratory viruses generally cause similar symptoms and the clinical presentation does not differentiate between the viruses [1,5]. Rate of progression to lower respiratory tract infection (RTI) varies between the different studies and ranges between 6.2–40% in LTRs [2,6]. Data on mortality associated with viral pneumonia in solid organ transplant (SOT) recipients are lacking, but a study on 98 LTRs found an attributable mortality of 5.1% [2].

## 2. Diagnosis

Historically, viral culture, direct fluorescent antibody (DFA) staining and serology were used to diagnose respiratory viral infections. Each method has its own shortcomings. Viral culture has a long turnaround time of 10 days for standard viral culture and two days for shell vial culture. DFA staining is available for only a limited number of respiratory viruses and requires expertise in interpreting the results. In addition, diagnoses based on serology have been useful only for epidemiological studies and not for diagnosing acute infection since acute- and convalescent-phase sera are needed [7]. With the advent of molecular strategies for diagnosis, other traditional methods have become obsolete. Nucleic acid testing (NAT) for respiratory viruses is now the gold standard for diagnosis and has a sensitivity of 72–100% [7]. In a study of 93 LTRs, 5/93 had respiratory viruses identified in bronchoalveolar lavage using viral culture and DFA staining, whereas 48/93 had respiratory viruses identified by NAT on the same samples [6]. Using multiplex NAT allows testing for several viruses simultaneously with a turnaround time of only 12–24 h [4]. Rapid antigen tests, allowing results within minutes, are clinically available for influenza and RSV only and suffer from low sensitivity [8].

## 3. Influenza Virus

### 3.1. Epidemiology and Risk Factors

Influenza virus is a single-stranded RNA virus of the *Orthomyxoviridae* family. There are multiple strains, although only influenza A and B are generally associated with disease in humans. Influenza viruses are seasonal, circulate mainly in the winter (months November to May in the Northern Hemisphere and May to October in the Southern Hemisphere), and cause a significant proportion of RTIs during that time [9,10]. The influenza attack rate depends on several factors, including age (with higher rates in children), likelihood of exposure, level of immunity (due to prior vaccination or disease), degree of immunosuppression and the nature of the epidemic [11]. A study evaluating incidence of influenza infections in 3569 SOT recipients between the years 1990 and 2000 calculated an incidence of 41.8, 2.8 and 4.3 per 1000 patient years in lung, liver and kidney transplants, respectively [12].

### 3.2. Clinical Presentation

Immunocompromised patients may not fit the classic definition of influenza-like illness. In a prospective multicenter study including 477 SOT recipients and 139 patients after hematopoietic stem cell transplant (HSCT) with confirmed influenza infection, the most common symptom was cough (85%), followed by fever (63%), rhinorrhea (48%), myalgia (40%), gastrointestinal symptoms (40%), sore throat (35%) and headache (30%) [13]. Immunocompromised individuals are also at increased risk for complications. These include viral pneumonia, bacterial superinfection, fungal coinfections, pericarditis and myocarditis, myositis, encephalopathy and encephalitis, etc. [14]. Compiling data from four studies with a total of 947 SOT recipients with influenza (both 2009 pandemic H1N1 and seasonal), hospitalization, intensive care unit (ICU) admission, pneumonia and mortality rates were 57–71%, 11–16%, 22–35% and 4–7.8%, respectively [13,15,16,17].

Risk factors associated with severe disease in multivariate analysis include older age, diabetes and use of mycophenolate mofetil. Univariate analysis also identified multiple comorbidities, use of antilymphocyte globulin in the past six months, lymphopenia, hypogammaglobulinemia, influenza A and nosocomial acquisition as risk factors for pneumonia or ICU admission [13,16].

### 3.3. Prevention

In the hospital setting, patients diagnosed with influenza should be placed under droplet precautions [18], as outbreaks in the hospital setting have been described [19]. However, the most important means for prevention is vaccination of the transplant recipient and close contacts. Only inactivated influenza vaccines should be given to transplant recipients. There is a theoretical risk of dissemination of virus contained in the live attenuated intranasal influenza vaccine, which is therefore contraindicated for SOT recipients [20]. The inactivated influenza vaccine has now been developed in a quadrivalent formulation and includes two A strains (H1N1 and H3N2) and two B strains. Previous trivalent formulations are being phased out. The American Society of Transplantation (AST) guidelines recommend vaccinating with an inactivated influenza vaccine as soon a one month post-transplant, acknowledging the fact that vaccine immunogenicity up to six months post-transplant can be poor [20]. Immunogenicity of influenza vaccine in SOT is variable, but generally lower compared to a non-immunocompromised population [21,22,23]. Still, influenza vaccine has been shown to decrease influenza infection rates, complications and mortality in the SOT population [13,19]. A large prospective multicenter study on 616 transplant recipients, mostly SOT, showed that vaccination in the same influenza season was associated with a reduction in odds for pneumonia (odds ratio (OR) 0.51, 95% CI 0.21–0.55) and admission to the ICU (OR 0.49, 95% CI 0.26–0.9) [13]. Another small study conducted during an influenza outbreak in a kidney transplant unit revealed that the unvaccinated population had significantly high rates of influenza infection (OR 37.5, 95% CI 2.7–507.5) and mortality (OR 6.7, 95% CI 2.3–18.9) [19].

In order to improve immune responses, several studies have evaluated different vaccination strategies in the SOT population and had variable outcomes. All studies assessed immune response and were underpowered for clinical outcomes such as confirmed influenza and influenza-like illness. A randomized controlled trial (RCT) on 60 kidney transplant recipients showed no difference in immune response to the adjuvanted and non-adjuvanted vaccines [24]. Another RCT on 212 SOT recipients compared high-dose intradermal vaccine with standard-dose intramuscular vaccine and also showed no difference between the two [25]. However, when comparing high-dose intramuscular vaccine to a standard-dose vaccine in 172 transplant recipients, the seroconversion rate to at least one strain was significantly higher (78.6% vs. 55.8%, *p* < 0.001) in the high-dose vaccine group [26]. Lastly, an RCT on 499 SOT recipients evaluating a booster vaccine strategy, giving two doses of standard-dose influenza vaccine five weeks apart, revealed significantly higher seroconversion rates in the booster vaccine group [27]. Therefore, 2019 guidelines by the American Society of Transplantation recommend that high-dose vaccination is the preferred strategy where available, although two doses of standard vaccine could also be used [20]. Several studies have shown that patients on mycophenolate have a worse antibody response compared to other immunosuppressive drugs [26,28], however there is no recommendation to withhold treatment around vaccination.

Another measure to prevent influenza infection would be using antiviral prophylaxis. Pre-exposure prophylaxis using low-dose oseltamivir was evaluated among 477 transplant recipients, mostly SOT, and demonstrated 80% efficacy against PCR-confirmed influenza (1.7% vs. 8.4%), without an increase in oseltamivir resistance [29]. Post-exposure prophylaxis has been evaluated in the non-immunocompromised population [30,31] and can be given in cases of exposure to influenza among transplant recipients who have contraindications to receiving the influenza vaccine or who are not expected to mount an immune response [10,14]. There is, however, some concern that these patients may develop antiviral resistance as they may already be infected at the time the prophylactic doses of oseltamivir are given. Consideration should be given to treating these patients with therapeutic-dose oseltamivir [9]. Guidelines published by The Infectious Diseases Society of America recommend that antiviral prophylaxis should be given in the case of a hospital outbreak to patients in the affected wards [14]. An outbreak is defined as two healthcare-associated cases diagnosed within 72 h in the same ward, and prophylaxis should be given for 14 days and at least 7 days after symptom onset in the last identified case. It should be noted, however, that this recommendation is based on studies performed during outbreaks in long-term care facilities [32,33]; data from hospital outbreaks, let alone among immunocompromised patients, are scarce [34]. Nevertheless, similar management can be pursued for an outbreak on a transplant ward.

### 3.4. Treatment

There are three groups of drugs approved for the treatment of influenza (Table 1 and Table 2). M2 inhibitors (amantadine and rimantadine) are not used today since they are inherently inactive against influenza B and circulating influenza A strains carry a high rate of resistance. The neuraminidase inhibitors (NAIs) are the group most commonly used, and include oseltamivir (oral), zanamivir (inhaled and intravenous), peramivir (intravenous) and laninamivir (available only in Japan and South Korea). Baloxavir was only recently approved and has a novel mechanism of action. It is a selective inhibitor of influenza cap-dependent endonuclease and is a single dose oral medication. It has been shown to be effective in uncomplicated influenza in the non-immunocompromised population [35]. Baloxavir may also be more effective against influenza B strains than NAIs [36].

There have been no trials comparing drugs, doses or treatment durations in the SOT population. According to trials comparing different NAIs in the immunocompetent population, no drug seems to be superior [37,38]. Observational studies in SOT recipients show that early antiviral treatment (within 24–48 h) is associated with a decrease in influenza complications and lower ICU admission rates [13,16,17]. There is also some evidence in transplant recipients [16] and the general population [39] that suggests patients who have symptoms > 48 h also benefit from treatment. Taking into consideration that transplant patients have prolonged shedding of influenza virus, the recommendation is that all symptomatic patients should be treated, irrespective of symptom duration [10]. However, an attempt should be made to start treatment empirically as soon as possible, usually before test results are available. Trials in immunocompetent patients failed to show superiority of high-dose (oseltamivir 150 mg twice daily) antiviral treatment [40,41]; however, some experts may choose to use high doses in cases of severe disease. Given the prolonged shedding in this population, some experts also extend treatment duration (e.g., 10-day oseltamivir treatment compared to the recommended 5-day regimen) in SOT recipients who are still symptomatic.

There are several investigational antivirals for influenza. Favipiravir is active against a wide spectrum of RNA viruses and as such can inhibit both influenza A and B viruses. Phase three studies have been carried out but data have not been published. Favipiravir may be promising as it seems to have low resistance rates and is synergistic when combined with oseltamivir. It is currently licensed only in Japan for use in influenza unresponsive or insufficiently responsive to current antivirals [42]. Pimodivir inhibits polymerase basic protein 2 in influenza A virus. Despite favorable initial reports [43], development has been discontinued after interim analysis of phase three studies showed it was unlikely to be of benefit.

Several monoclonal antibodies targeting various hemagglutinins of influenza virus have been developed [44]. These were tested in phase two studies as monotherapy or in combination with antiviral drugs, showing mixed results [45,46].

Immunocompromised patients are at increased risk for antiviral resistance due to prolonged viral replication combined with antiviral exposure, which is sometimes subtherapeutic (as in post-exposure prophylaxis) [14]. As stated, M2 inhibitors are not recommended due to the high resistance rate in the currently circulating influenza A strains. Resistance to NAIs for seasonal influenza is uncommon and occurs predominantly in the A/H1N1 strain [47,48]. The most common mutation is H275Y, found in A/H1N1, however, this mutation still maintains susceptibility to zanamivir [10,14]. The most prevalent mutation in A/H3N2 is R292K, which it confers reduced susceptibility to both oseltamivir and zanamivir [48]. Several case reports of emergent resistance during NAI use have been published, mainly among patients with hematological malignancies or after stem cell transplants [49,50]. NAI resistance should be suspected in a transplant patient with a prolonged illness and persistent viral replication, or in those who developed influenza while on or shortly after receiving low dose antivirals. Treatment options include changing to a different NAI, changing to a different antiviral class, or combination antivirals. Resistance has also been documented in 9.7% of patients treated with baloxavir; this was associated with prolonged shedding of the virus and longer time to alleviation of symptoms [35].

## 4. Respiratory Syncytial Virus

### 4.1. Epidemiology and Risk Factors

RSV is a single-stranded RNA virus of the *Pneumoviridae* family (formerly *Paramyxoviridae*) and has two strains: RSV-A and RSV-B. It is a seasonal virus with peak incidence in the winter and spring [2,51] and circulates mainly among young children, who are a significant source of transmission [9]. In LTRs, RSV accounts for 2.4–6.2% of respiratory viruses identified in upper and lower respiratory tract specimens [1,2,6] and is a cause of significant morbidity and mortality due to the development of lower RTI [10,52,53,54]. Risk factors for lower RTI and mortality in the SOT population are poorly defined, but include young children (less than two years old), recent transplant, lung or multivisceral transplant and recent rejection [52].

### 4.2. Clinical Manifestations

The clinical presentation of RSV is similar to other respiratory viruses, as it commonly presents with fever, cough, dyspnea and rhinorrhea [1,5,55]. Compared to other respiratory viruses, RSV more frequently causes lower RTI, including bronchitis, bronchiolitis and pneumonia [5]. This is especially true for LTRs, where lower RTI rates can be as high as 72% [2,55].

### 4.3. Prevention

Hospitalized patients should be placed under contact precautions [18] as RSV droplets form large particles and are transmitted by contact. The American Academy of Pediatrics recommends consideration of palivizumab prophylaxis during the RSV season in children under 24 months of age who are severely immunocompromised, acknowledging that this recommendation is not evidence based [56]. A survey conducted among 67 pediatric SOT centers in the US revealed that approximately half of these centers use palivizumab prophylaxis [57]. Since prophylaxis is given on a monthly basis during the RSV season and dosing is weight-based, costs of treatment in adults are extremely high. Combined with the fact that there are no data to support this practice, palivizumab is generally not given to adult SOT recipients. Current efforts in the area of monoclonal antibody development are directed at developing antibodies with extended half-lives which can potentially lower costs [58]. Nirsevimab is a novel monoclonal that targets an epitope of the RSV fusion protein. Due to its extended half-life, it can be given once per season and has shown favorable results in a recently published randomized trial conducted on preterm infants [59].

Vaccine development was put on hold after a trial from the 1960s demonstrated worsening disease that was associated with death of two infants. There are currently multiple vaccines under development, including live-attenuated, viral-recombinant, subunit and nanoparticle-based. These vaccines are recommended for elderly persons, pregnant women or the pediatric population [58]. The most advanced is ResVax, which is a nanoparticle-based vaccine; however, a recent phase three trial of this vaccine in pregnant women did not reduce RSV infections in infants after birth [60].

### 4.4. Treatment

A survey conducted in 11 transplant centers in the United States revealed differences in treatment regimens in lung transplant compared to other organs. Among 10 lung transplant centers all treat lower RTI with ribavirin and three centers add intravenous immunoglobulin (IVIG), whereas only 6/10 centers treat upper RTI with ribavirin and none give IVIG. Among 11 non-lung transplant centers 7/11 treat lower RTI with ribavirin and only one center adds IVIG, whereas in upper RTI no center gives treatment [61]. Data on treatment for RSV infection in SOT recipients are limited to case series in lung transplants (Table 1 and Table 2). Data in the HSCT population are slightly more robust and include larger case series, prospective cohorts and one small RCT [62]. In a combined analysis of trials done in HSCT, ribavirin treatment was associated with reduced progression of upper RTI to lower RTI and decreased mortality among patients with lower RTI. Patients who received a combination of ribavirin and an immunomodulator (IVIG, RSV-IVIG or palivizumab) had significantly lower mortality when treated for lower RTI, and a non-significant reduction in progression to lower RTI [63]. As most of the studies included in this analysis used inhaled ribavirin, data on systemic ribavirin (oral and intravenous) did not reach statistical significance.

As mentioned, data in lung transplants are of low quality. One case series published in 2011 compared 38 patients treated with oral ribavirin with 29 patients that received only supportive care and demonstrated that oral ribavirin treatment was associated with improvement in graft function and reduction in bronchiolitis obliterans syndrome [64]. On the other hand, a study on 10 lung transplant recipients with lower RTI due to RSV showed good outcomes with mainly supportive care [65]. Other case series also support the use of oral ribavirin, which demonstrated good outcomes in both comparative [66] and non-comparative cohorts [67]. Administering inhaled ribavirin is challenging as it needs to be given in negative-pressure rooms due to its teratogenicity. It is also extremely expensive, making oral ribavirin a more attractive option. Notably, systemic ribavirin is also associated with significant side effects, mainly hemolytic anemia, leukopenia, neuropsychiatric symptoms, and is also teratogenic.

Presatovir is a new antiviral with specific anti-RSV activity that inhibits fusion of the virus with the host cells. Two phase 2b RCTs have been conducted in HSCT (one each for upper and lower RTI) [68,69] and one RCT was conducted in LTRs [70]. All trials failed to show significant improvements in clinical and virologic outcomes in the presatovir group, except for a possible decrease in progression to lower RTI in lymphopenic HSCT recipients. Additionally, a significant portion (20%) of patients in the presatovir group developed resistance [68].

Several new drugs targeting RSV are currently under development, however none have reached phase three trials [58]. These drugs can be divided into fusion inhibitors (RV521, AK0529/ziresovir) and replication inhibitors (PC786, EDP-938).

## 5. Human Metapneumovirus

HMPV is a single-stranded RNA virus of the *Pneumoviridae* family, closely resembling RSV. Its seasonality also follows that of RSV, with most cases identified in the winter and spring [71]. Studies conducted on LTRs identified HMPV in 3.6–6.8% of positive respiratory samples [1,2,6,72]. Most of these patients had symptoms of RTI, and in one study cohort, 8/18 (44%) had a lower RTI [2].

Ribavirin has in vitro activity against HMPV. However, data derived from HSCT recipients do not demonstrate a mortality benefit with antivirals or immunomodulators [73]. Data in SOT are based solely on lung transplants and are limited to small case series. In a study that included 139 LTRs with infections due to either RSV, HMPV or PIV, ribavirin (mostly given using oral preparation) was associated with significantly less chronic lung allograft disease (CLAD, OR 0.24, 95% CI 0.1–0.59) [71]. Two other small series of 15 and 19 lung transplant recipients with HMPV infection demonstrated favorable outcomes on graft function among patients treated with ribavirin with or without steroids [74,75]. Although these data may suggest some benefit from ribavirin in lung transplants, controlled studies are lacking, and current treatment is primarily based on supportive care. Prevention is mainly based on infection control practices, including implementation of contact precautions in hospitalized patients [18].

## 6. Parainfluenza Virus

Parainfluenza virus (PIV) is a single-stranded RNA virus of the *Paramyxoviridae* family. There are four serotypes of PIV (1–4); serotype 3 is the most common and shows no seasonality and has been associated with outbreaks, whereas serotypes 1 and 2 appear in the fall and winter [4,71]. As with other respiratory viruses, PIV infection was mainly studied in LTRs, where it accounts for 3.6–20.9% of the respiratory viruses isolated [1,2,6]. PIV infection in LTRs is associated with a high rate of symptomatic disease and lower RTI [2]. In a study on 24 LTRs with PIV infection, mostly PIV3, 21% experienced respiratory failure [76].

Utility of antivirals for PIV infection in SOT is unknown. A systematic review on antiviral treatment for PIV infection in HSCT recipients showed no benefit in this population [77]. Studies in SOT recipients are limited to small case series describing the use of ribavirin with and without immunomodulators in LTRs with mixed viral infections (PIV and RSV with/without HMPV) [71,78,79]. These studies showed mixed responses to the treatments used. DAS 181 is a novel, inhaled sialidase that cleaves sialic acid from the host’s respiratory epithelium, thus preventing attachment and entry of viruses such as PIV and influenza virus into respiratory cells [80]. DAS 181 was evaluated in an RCT among immunocompromised patients with lower RTI secondary to PIV infection. Although there was a trend towards better outcome (composite of clinical stability and survival) in the DAS 181 group, this did not reach statistical significance. A post-hoc analysis suggested significantly improved outcomes in the severely immunocompromised subgroup [80]. DAS 181 is not currently FDA approved. Thus, the mainstay of treatment for PIV infection is supportive care and prevention in the hospital setting is based on adherence to contact precautions [18].

## 7. Rhinovirus

Rhinoviruses are single-stranded RNA viruses that are members of the *Picornaviridae* family, which is part of the *Enterovirus* genus. Serotypes A–C circulate year-round and are the predominant cause for the common cold [2,4]. As such, these viruses are isolated most frequently in respiratory samples taken from immunocompetent patients as well as SOT recipients. In studies among LTRs evaluating respiratory viruses found in respiratory samples by PCR, rhinoviruses accounted for 41.8–61.6% of the positive samples [1,2,6]. One study compared infection rates in 36 LTRs with a cohort of 235 immunocompromised and immunocompetent patients, and showed a higher infection rate among LTRs (41.7% vs. 14.5%, *p* < 0.001) [81]. Rhinoviruses are frequently found as part of coinfection with other viruses or bacteria, rendering the relative part of rhinoviruses unknown [4,5]. Symptoms of rhinovirus infection are usually those of the common cold, although there have been case reports of lower RTI in LTRs [82]. Another study suggested a correlation between higher viral load and more symptoms [83]. Treatment is based on supportive measures and prevention in the hospital setting mandates droplet precautions [18].

## 8. Coronaviruses

CoVs are single-stranded RNA viruses of the *Coronaviridae* family. Established human CoVs (229E, NL63, OC43, HKU1) cause upper RTI, whereas other CoVs (severe acute respiratory syndrome (SARS)-CoV1, Middle Eastern respiratory syndrome (MERS)-CoV, SARS-CoV-2) are associated with outbreaks of severe respiratory disease. This review will not cover infections caused by SARS-CoV-2.

Human coronaviruses are second only to rhinoviruses for prevalence among LTRs, accounting for 12.4–17.8% of the positive samples [1,2,6]. Symptoms are generally similar to other respiratory viruses. A study on 85 immunocompromised and 1152 immunocompetent children demonstrated a similar rate of lower RTI in the two groups (22% and 26%, respectively); however, the immunocompromised group had a significantly higher rate of severe lower RTI [84]. Studies done on LTRs [2] and HSCT recipients [85] showed comparable rates of lower RTI.

SARS-CoV1 infection emerged in southern China in late 2002 [86] and was associated with high rates of lower RTI and mortality as high as 20% [87]. In-vitro studies identified ribavirin and interferon as active against the virus [87], although no clear clinical benefit of ribavirin was seen. Case series and a small RCT suggested better outcomes with interferon and high-dose steroids in conjunction with supportive care [88,89]. SARS-CoV1 infection was described in two transplant recipients; the first was a liver transplant patient that was exposed in the healthcare setting and infected several healthcare workers. He was treated with ribavirin, but eventually succumbed [90]. An additional fatal case of a lung transplant was also described [91]. Thanks to strict infection control practices the outbreak was controlled and there were no more cases of SARS-CoV1.

MERS-CoV infection was first identified in Saudi Arabia [92] and is associated with severe respiratory illness and mortality rates as high as 50% [93]. Human to human transmission is associated with healthcare settings, and in one of the cohorts studied, 25% of the people infected were healthcare workers [94]. There is also ongoing zoonotic transmission from camels [95]. Two cases in kidney transplant recipients have been described, and only one of the two recovered [96]. Treatment options used in published case reports include ribavirin, interferon and steroids [93,94]. MERS-CoV still causes sporadic infections, mainly in the Middle East [95].

## 9. Adenovirus

### 9.1. Epidemiology

Adenovirus is a double-stranded DNA virus of the *Adenoviridae* family that has seven subgroups (A-G) and almost currently known 90 serotypes [97]. Adenoviruses establish latency in lymphoid tissue; thus, infection can represent reactivation or de novo community acquisition [98]. Adenoviruses show no seasonal variability [9,99] and have been associated with institutional outbreaks [100,101].

Adenoviruses are known to cause viremia without obvious symptoms; therefore, differentiating infection and disease may be more appropriate [102]. In a study of 263 SOT recipients, adenovirus in blood was checked at regular intervals during the first year post-transplant. As much as 7.2% (19/263) developed viremia; however, only 4/19 (21%) were symptomatic [103]. Rates of adenovirus infection differ with age and the transplanted organ. Rates are higher among children, probably because they are more likely to be non-immune, and in intestinal transplantation, presumably because of the higher amount of lymphoid tissue in the allograft and greater immunosuppression [99,102,104].

### 9.2. Clinical Manifestations

Adenovirus infection can manifest with conjunctivitis, upper RTI, lower RTI, hemorrhagic cystitis, pyelonephritis, hepatitis and enterocolitis, and has a predilection for the transplanted allograft [99,102]. In a kidney transplant recipient with fever of unknown origin and rise in creatinine, adenovirus should be considered as a cause of pyelonephritis [105]. Mortality can be as high as 50% for adenoviral pneumonia and 80% for disseminated adenoviral infection [102]. Although in most cases there are no long term sequelae [103,106], development of bronchiolitis obliterans has been described in lung transplant recipients [107].

### 9.3. Prevention

Adenovirus infections are transmitted by respiratory droplets, direct conjunctival inoculation, person-to-person contact, infected fomites and the fecal–oral route. Therefore, prevention in the hospital setting is based on maintaining droplet and contact precautions [18]. Brincidofovir is an orally bioavailable lipid conjugate of cidofovir that lacks the nephrotoxicity associated with cidofovir. It was tested in a phase two RCT for preemptive treatment of adenovirus viremia in HSCT recipients and showed benefits [108]; however, it is not FDA approved and the appropriate dose for treatment of adenovirus is not established.

### 9.4. Treatment

Data on treatment modalities for adenovirus disease are derived from case reports and small case series. Aside from supportive care, treatment options include reduction of immunosuppression, cidofovir, brincidofovir (investigational), IVIG and adenovirus-specific cytotoxic T lymphocytes (investigational). Earlier case reports described favorable outcomes when combining cidofovir with reduction of immunosuppression [106,109,110,111]. Contemporary data, the largest series being 13 liver transplant recipients, show promising results with the use of brincidofovir as well as with its use in combination with the reduction of immunosuppression [108,112,113,114]. Some may use IVIG, usually in addition to an antiviral drug [110]. Using virus-specific cytotoxic T lymphocytes for the treatment of cytomegalovirus and Epstein–Barr virus infections in HSCT recipients shows great promise. Data regarding adenovirus infections are scarce in HSCT [115,116] and still lacking in SOT.

## 10. Bocavirus

Bocavirus is a single-stranded DNA virus of the *Parvoviridae* family. It is rarely isolated from respiratory specimens, and in positive specimens, there is often co-infection with other respiratory viruses [5,9]. Among LTRs, bocaviruses were isolated from only 0.5–1% of the positive respiratory samples [1,2]. Additionally, similar to the closely related parvovirus B19, bocaviruses are known for their viral persistence [9]. This, combined with the high co-infection rate, makes their true contribution to RTIs unknown.

## 11. KI and WU Polyomaviruses

KI and WU polyomaviruses are double-stranded DNA viruses of the *Polyomaviridae* family that were discovered in 2007. Studies in HSCT recipients suggested a higher frequency of infection with these viruses [4,117]. A study done in kidney transplant recipients identified KI and WU polyomaviruses in 14.3% and 9.1% of respiratory specimens, respectively [118]. However, it is still unclear whether this has any clinical significance

## 12. Respiratory Viruses and Rejection in Lung Transplantation

As previously mentioned, LTRs are at increased risk for RTIs in general and specifically lower RTIs [4]. This may be related to continuous contact of the allograft with the environment, impaired mucociliary clearance, impaired cough reflex and a relatively greater immunosuppression compared to other organ transplants. Numerous studies have evaluated the association between respiratory viral infection and acute rejection or CLAD/bronchiolitis obliterans syndrome (BOS) showing conflicting data [6,119,120,121,122]. A systematic review and meta-analysis published in 2011 demonstrated no association between respiratory viral infection and acute rejection, although only four studies were included in the analysis. It also showed a non-significant trend towards association with BOS, but that was limited by small numbers [123]. A study on 100 LTRs, half with RTIs and half without, showed significantly higher rates of acute rejection (16% vs. 0%) and biopsy-proven bronchiolitis obliterans (10% vs. 0%) among those with respiratory infections [122]. Associations have also been reported for specific respiratory viruses, including influenza [13,124], HMPV and RSV [64,72], PIV [76] and adenovirus [107]. It seems, however, that there is no definitive association between rhinoviruses and CoVs and rejection or CLAD [72,125]. Treatment with ribavirin was associated with lower rates of CLAD/BOS in LTRs with RSV, HMPV and PIV infections, as shown in a study on 139 LTRs where ribavirin was associated with a lower risk of CLAD (OR 0.24, 95% CI 0.1–0.59) [71].

## 13. Summary

Respiratory viruses are a significant cause of morbidity and mortality among SOT recipients. With the introduction of molecular diagnostic methods, they are detected at a greater frequency and diversity. Effective therapies are available only for influenza, and also to some extent for RSV infection; however, new drug classes show some promise. Preventive measures are also lacking, as vaccination is only available against influenza at this time. Given the severe implications respiratory viral infections have on the immunocompromised population, development of new antivirals and vaccines is needed.

## Figures and Tables

**Table 1:** Treatment and preventive measures for respiratory viral infections.

	Treatment *	Prevention
Influenza Virus	Neuraminidase inhibitors	Droplet precautions Vaccination Pre-exposure and post-exposure antiviral prophylaxis
Respiratory Syncytial Virus	Ribavirin ± IVIG/steroids	Contact precautions Palivizumab for age < 2 years
Parainfluenza Virus	Ribavirin ± IVIG/steroids? DAS 181-investigational	Contact precautions
Human Metapneumovirus	Ribavirin ± IVIG/steroids?	Contact precautions
Rhinovirus	None	Droplet precautions
Human Coronaviruses	None	Standard precautions
SARS-CoV1, MERS-CoV	Ribavirin, interferon, steroids	Airborne & contact & droplet precautions
Adenovirus	Cidofovir, brincidofovir (investigational), IVIG	Droplet & contact precautions
Bocavirus	None	Standard precautions

* Reduction of immunosuppression is recommended for all severe respiratory illnesses. SARS-CoV1—severe acute respiratory syndrome- coronavirus 1; MERS-CoV—Middle Eastern respiratory syndrome- coronavirus; IVIG—intravenous immunoglobulin.

**Table 2:** Dosing of commonly used antivirals.

	Treatment Dose	Prophylactic Dose
Oseltamivir (oral)	75 mg q12h (5 days)	75 mg q24h
Zanamivir (inhaled)	10 mg q12h (5 days)	10 mg q24h
Peramivir (IV)	600 mg once	NR
Baloxavir (oral)	40 mg (<80 kg), 80 mg (>80 kg)	40 mg (<80 kg), 80 mg (>80 kg)
Ribavirin (IV/oral)	LD 600 mg then 200 mg q8h for 1 day then 400 mg q8h. Can increase to maximum 10 mg/kg q8h.	NR
Cidofovir (IV)	1 mg/kg 3 times a week, or 5 mg/kg once a week for 2 weeks and then every 2 weeks Add probenecid and hydration	NR

Dose is for adults with normal renal function. IV—intravenous; LD—loading dose; NR—not relevant; kg—kilogram.

## Data Availability

Not applicable.
